# Efficient Strategy to Synthesize Tunable pH-Responsive Hybrid Micelles Based on Iron Oxide and Gold Nanoparticles

**DOI:** 10.1021/acs.langmuir.4c01318

**Published:** 2024-05-20

**Authors:** Raúl Gimeno-Ferrero, Javier Rodríguez de Jesús, Manuel Pernia Leal

**Affiliations:** Departamento de Química Orgánica y Farmacéutica, Facultad de Farmacia, Universidad de Sevilla, c/Profesor García González, 2, 41012 Sevilla, Spain

## Abstract

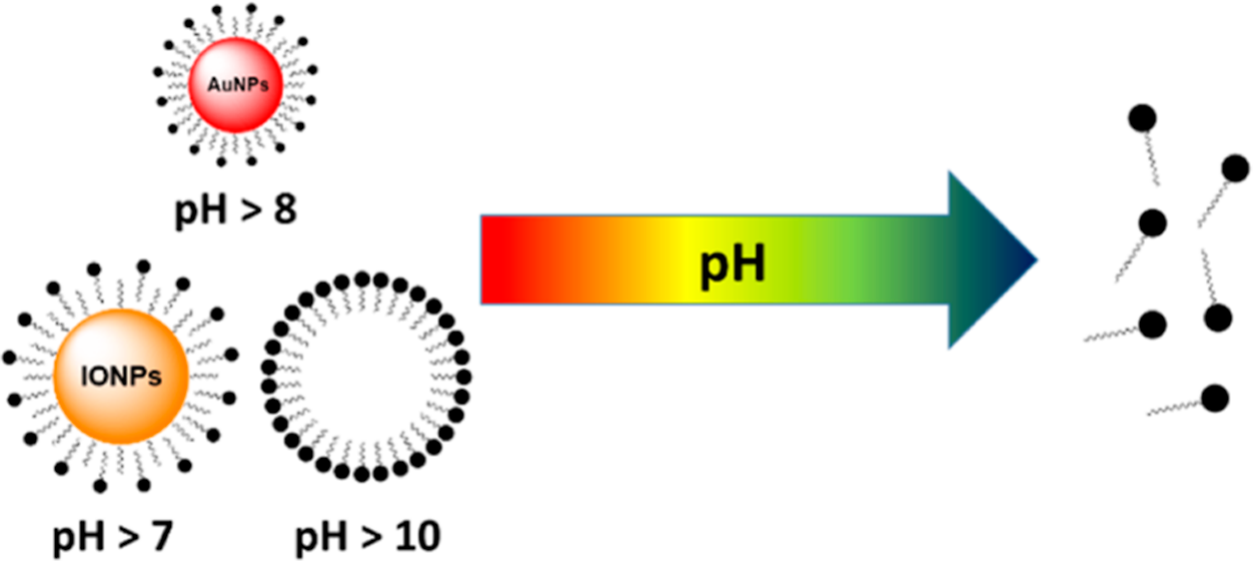

The preparation of multifunctional nanomaterials based on inorganic nanoparticles with organic materials has emerged as a promising strategy for the development of new nanomedicines for in vitro and in vivo biomedical applications. Here, we synthesized pH-responsive hybrid inorganic micelles by combining a novel pH-responsive amphiphilic molecule with hydrophobic payloads. This amphiphile was synthesized in a one-pot reaction and self-assembled readily into micelles under acidic pH conditions. In the presence of hydrophobic NP payloads such as AuNPs or IONPs, the amphiphile self-organized around them through hydrophobic interactions, resulting in the formation of colloidally stable hybrid micelles. The size of the hydrophobic NPs determined the pH-response of the inorganic hybrid micelles, which is tuned from pH 7 to 11 for our pH-responsive amphiphilic molecule. This achievement represents a novel approach for the synthesis of tunable pH-responsive hybrid micelles based on inorganic NPs for biomedical imaging, hyperthermia treatment, and also drug delivery nanosystems.

## 1 Introduction

Inorganic hybrid nanomaterials have been attracting a great interest in the past decade in the medical research field as promising agents for the diagnosis and treatment of multiple diseases.^1−6^ This class of materials are mainly made by the combination of inorganic nanoparticles (NPs) with organic materials.^7−12^ Sphere- and cube-shaped iron oxide-based NPs in different sizes are the most studied colloidal magnetic NPs due to their excellent characteristics as magnetic resonance imaging contrast agents and also as heat mediators in magnetic hyperthermia.^13−15^ AuNPs and gold-based NPs are also intensively studied as bioimaging and biosensing agents.^3^ On the other hand, organic materials such as micelles, liposomes, dendrimers, and polymers are combined with inorganic NPs to render hybrid nanomaterials highly soluble under physiological conditions without altering their initial properties.^16^ For instance, in the Pellegrino group, we have efficiently developed robust methods to prepare multifunction hybrid nanobeads using poly(maleic anhydride-alt-1-octadecene) as the amphiphilic polymer. These nanobeads can be made of one type of NPs or a mixture of diverse functional inorganic NPs, such as IONPs and rare earth NPs, to obtain multimodal imaging agents.^17^ Lipid-based formulations, such as liposomes and micelles, are other important and well-known formulations for the preparation of hybrid nanomaterials with high potential to enter in clinical trials or even to be approved for clinic.^18,19^ Several groups have reported effective synthesis of magneto liposomes for in vivo applications.^20−23^ The utilization of liposomes enables the incorporation of a sufficient amount of magnetic NPs within the membrane or the lumen of the liposomes. On the other hand, micelles have been reported as promising nanoformulations to carry inorganic NP payloads due to their smaller sizes than other lipid-based nanomaterials, ease of chemical modifications of the amphiphiles, and excellent pharmacokinetics.^24−26^ The incorporation of stimuli-response functionality into the organic material is another key factor in the preparation of hybrid nanomaterials.^27−30^ These functions are triggered in response to external stimuli, such as pH, temperature, or redox conditions. Overall, pH change is one of the most widely investigated stimuli for biomedical applications. This pH variation has led to a large number of studies on pH-responsive nanomaterials.^31^ In this sense, lipid nanocarriers, such as pH-responsive micelles,^18,32−34^ represent versatile and dynamic nanomaterials that self-assembled from amphiphilic molecules and disassembled under specific pH conditions. This property makes them excellent nanocarriers with a wide variety of payloads in medical applications.

Herein, we report a straightforward method for the preparation of tunable pH-responsive micelles based on tertiary amine-derived amphiphilic molecules and hydrophobic payloads. The size of the hydrophobic payloads influences the pH-responsiveness and the stability of the hybrid micelles. Micelles with larger hydrophobic payloads such as NPs with a diameter of 18 nm disassemble at pH 7, whereas micelles with smaller hydrophobic NPs with a diameter of 9 nm disassemble at higher pH values, specifically, pH 8. The absence of any payload elevates the stability of the micelles in water at pH > 10.

## 2 Materials and Methods

### 2.1 Chemicals and Solvents

Chemicals and solvents were purchased from commercial suppliers (Merck and Fisher Scientific) and used as received. Tetrachloroauric(III) acid trihydrate, iron(III) chloride, sodium oleate, oleic acid, oleylamine, oleyl alcohol, 1-octadecene, 4-(dimethylamino)butanoic acid, oxalyl chloride, (*S*)-camptothecin, 2-(*N*-morpholino)ethanesulfonic acid (MES), hydrochloric acid, sodium hydroxide, sodium chloride, sodium phosphate monobasic, sodium phosphate dibasic, sodium sulfate, and potassium chloride were used. Solvents such as toluene, ethanol, acetone, hexane, chloroform, dichloromethane, and tetrahydrofuran were used. These solvents were anhydrous and HPLC grade. Milli-Q water (18.2 MΩ, filtered with a filter pore size of 0.22 μM) was from Millipore. Phosphate-buffered saline (PBS) 1× contains 137 mM NaCl, 2.7 mM KCl, 10 mM Na_2_HPO_4_, and 1.8 mM KH_2_PO_4_. PBS was adjusted to pH 7.2 with diluted solutions of HCl and NaOH. The monitoring of the organic synthesis was performed by thin-layer chromatography (TLC) on a sheet of aluminum coated with silica gel 60 F254 purchased from Merck. TLCs were revealed with a solution of 5% phosphomolybdic acid/EtOH. Silica gel 60 (0.04–0.063 mm) for flash chromatography from Merck was used. The chromatographic column was eluted with a positive pressure of air, and eluents are given as volume-to-volume ratios (v/v).

### 2.2 Measurement Techniques

#### 2.2.1 Nuclear Magnetic Resonance (NMR)

Nuclear magnetic resonance (NMR) spectra were recorded on a BRUKER AMX-500 apparatus. Deuterated chloroform was used and indicated in parentheses for the compound. Chemical shift values (δ) refer to tetramethylsilane used as the internal reference.

#### 2.2.2 High-Resolution Mass Spectrometry (HRMS)

High-resolution mass spectrometry (HRMS) was recorded on a Kratos MS-80-RFA apparatus by using electrospray ionization (ESI) in a positive mode.

#### 2.2.3 Dynamic Light Scattering (DLS)

Dynamic light scattering (DLS) was recorded by means of a Malvern Nano ZS90 instrument equipped with a 4.0 mW HeNe laser (633 nm). The measurements were carried out on a cell type: ZEN0040 disposable cuvette cell type, setting a refractive index of 2.30 for the iron oxide NPs, 0.20 for the gold NPs, and 1.40 for the micelles without inorganic NPs with 173° Backscatter (NIBS default) as angle of detection. The measurement duration was set as automatic, and three was the number of measurements. As the analysis model, the normal resolution was chosen. For the size distribution measurement, the intensity mean was selected. In order to measure the hydrodynamic size versus pH dependency, each pH value was adjusted by adding the necessary amount of hydrochloric or sodium hydroxide to a small amount of the micelles (approximately 1 mL of the micelle solution at a concentration of 1 mg/L micelles with or without inorganic NPs). For each of these measurements, a fresh aliquot of micelles was employed.

#### 2.2.4 Transmission Electron Microscopy (TEM)

Transmission electron microscopy (TEM) was measured on an HR Fei Talos 200× microscope operated at an accelerating voltage of 100 kV. The samples were prepared by drop casting a solution of the sample onto a carbon-coated copper grid followed by removing the liquid by evaporation under ambient conditions and without adding any staining. The mean sizes were calculated on an average of hundred NPs measured.

#### 2.2.5 UV–Visible Absorption Spectra

UV–visible absorption spectra were measured by using a Varian Cary 300 UV–vis spectrophotometer. The samples were diluted 1:20 in the corresponding solvent using a quartz cuvette with 1 cm light path.

#### 2.2.6 Elemental Analysis

Elemental analysis was carried out via inductively coupled plasma (ICP) atomic emission spectroscopy on a SpectroBLUE instrument. ICP samples were prepared by incubating overnight 25 μL of inorganic NPs in 2.5 mL of aqua regia. The mixtures were diluted with Milli-Q water to 25 mL.

#### 2.2.7 Fluorescence Measurements

Fluorescence measurements were carried out on a PerkinElmer LS55 fluorescence spectrometer with excitation wavelength of 370 nm, and the emission spectrum was recorded from 390 to 600 nm for the drug release studies.

#### 2.2.8 Magnetization-Field M–H Curves

The magnetization-field M–H curves were obtained using a Quantum Lot-physical property magnetic system (PPMS) instrument at room temperature.

#### 2.2.9 Small-Angle X-ray Scattering (SAXS)

Small-angle X-ray scattering (SAXS) measurements were carried out in a Bruker D8 DISCOVER diffractometer. The data were treated using RAW software, and pair distribution functions were obtained using GNOM.^35^

### 2.3 Synthesis of Oleyl 4-(dimethylamino) Butanoate (**2**)

To a solution of 4-(dimethylamino) butyric acid hydrochloride (1.19 mmol) in dry DCM (5 mL) in a 50 mL round-bottom flask at room temperature under an argon atmosphere was added oxalyl chloride (2.39 mmol). The reaction mixture was refluxed for 4 h. The DCM and oxalyl chloride were removed under reduced pressure. The mixture was dissolved in dry DCM (5 mL) and under an argon atmosphere was added oleyl alcohol (1.19 mmol). The reaction mixture was stirred overnight, and then DCM was evaporated under reduced pressure. The solid was filtered and purified by flash chromatography (DCM/MeOH, 15:1) to obtain 320 mg of the desired compound **2** (64% yield) as a white solid; Rf = 0.66 (DCM/MeOH, 9:1).

^1^H NMR (500 MHz, CDCl_3_): δ (ppm): 5.38–5.29 (m, 2H), 4.04 (t, *J* = 6.7 Hz, 2H), 2.42 (t, *J* = 7.6 Hz, 2H), 2.34 (t, *J* = 7.3 Hz, 2H), 2.32 (s, 6H), 2.03–1.97 (m, 4H), 1.85 (q, *J* = 7.4 Hz, 2H), 1.59 (q, *J* = 6.8 Hz, 2H), 1.36–1.2 (m, 24H), 0.86 (t, *J* = 6.8 Hz, 3H).

^13^C NMR (125 MHz, CDCl_3_): δ (ppm): 173.9, 130.2, 129.79, 64.65, 58.52, 44.86, 31.91, 31.86, 29.77, 29.74, 29.70, 29.66, 29.53, 29.42, 29.24, 29.22, 28.63, 27.23, 27.19, 25.93, 22.69, 22.29, 14.11.

HRMS (ESI+): *m*/*z* predicted for C_24_H_47_NO_2_: [M + H]^+^ 382.38 found 382.3673 (∼1.826 ppm).

### 2.4 Synthesis of Fe_3_O_4_ Nanoparticles

The preparation of Fe_3_O_4_ NPs of 18 nm was prepared with some modifications according to the procedure reported in a previous work.^36^ Briefly, 1 mmol iron oleate, 0.5 mmol oleic acid, and 10 g of 1-octadecene were mixed and heated to reflux for 1 h under a flow of argon. Then, the reaction was washed several times with a mixture of ethanol, acetone, and isopropanol as precipitating agents and centrifuged followed by redispersion in toluene.

### 2.5 Synthesis of Gold Nanoparticles

The synthesis of the Au NPs of 9 nm was prepared following the procedure already reported.^37^ 0.3 mmol HAuCl_4_·3·H_2_O in 1 mL of oleylamine was added rapidly to 5 mL of oleylamine heated at refluxed under a flow of argon. The reaction mixture was heated with magnetic stirring for 1.5 h. Then, the reaction was washed with ethanol to precipitate the NPs and centrifuged for 5 min at 4000 rpm. The particles were redispersed in hexane.

### 2.6 Determination of the Critical Micelle Concentration (CMC) of Oleyl 4-(dimethylamino) Butanoate

The determination of the CMC of **2** was performed following the method described elsewhere.^38^ Fluorescence measurements were carried out on a Hitachi F-2500 fluorescence spectrophotometer with excitation wavelength of 334 nm, and the emission spectrum was recorded from 350 to 500 nm. To a series of tertiary amine-derived amphiphile solutions in water with different concentrations (300 μL, from 1 to 10^–3^ mM) was added a solution of pyrene in water (300 μL, 0.7 μM). The mixtures were shaken for 30 min. The fluorescence intensities at wavelengths of 372 (I1) and 383 nm (I3) were extracted from the spectra, and the ratio between them (I3/I1) was plotted vs the amphiphile concentration. The intersection of the two lines determined the CMC.

### 2.7 Synthesis of Hybrid Inorganic Micelles

To a solution of amphiphile **2** (5 mL, 1 mg/mL) was added a colloidal suspension of hydrophobic NPs, IONPs, or AuNPs (100 μL, [NP] = 10^–9^ M). Then, the formation of the micelles was performed by following different protocols:

A Evaporation at room temperature and pressure. The mixture was left in an uncapped vial until the evaporation of the apolar solvent (ca. 12 h).
B Shaking. The mixture was shaken at 230 rpm in a vortex shaker until the evaporation of the apolar solvent (ca. 2 h).
C Sonication bath. The mixture is sonicated in a sonication bath for 30 min.
D Probe sonicator. The mixture is sonicated with a probe sonicator during 30 min with an amplitude of 30% and power of 55 W.

### 2.8 In Vitro Release Studies of (S)-Camptothecin

First, camptothecin (CPT) was encapsulated into micelles. In a glass vial, 5 mg of amphiphilic molecule **2**, 0.1 mg of CPT, and 5 mL of water were added. The mixture was sonicated using a probe sonicator, following the same conditions described above. After that, the aqueous mixture was centrifuged at 1500 rpm, and the supernatant, which contains the CPT-loaded micelles, was collected for the release studies. A solution of CPT-loaded micelles was dialyzed at room temperature under stirring against 2 L of the corresponding buffer (MES or PBS). After defined time lapses, an aliquot was taken, and the fluorescence emission spectra were recorded upon excitation at 370 nm.

## 3 Results and Discussion

### 3.1 Synthesis and Characterization of pH-Responsive Micelles

**Scheme 1:**
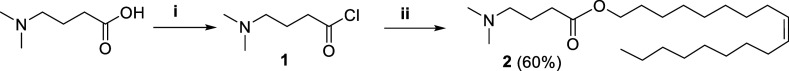
Synthesis of Oleyl 4-(dimethylamino) Butanoate

The synthesis of the pH-responsive micelles was performed following a previous reported method to prepare micellar nanocarriers based on the use of oleic acid as lipophilic tail.^39^ Here, we conducted a one-pot synthesis of a new micelle precursor consisting of the incorporation in the hydrophobic chain of a tertiary amine-derived moiety as the polar and pH-responsive head (Scheme 1). First of all, the corresponding acyl chloride **1** was synthesized from the 4-(dimethylamino)butanoic acid in the presence of oxalyl chloride. Then, after the evaporation of the volatiles, it was added oleyl alcohol to the mixture to successfully yield the desired compound oleyl 4-(dimethylamino) butanoate.

The NMR characterization showed the ester formation between the tertiary amine moiety and the lipophilic tail, identifying, in the ^1^H NMR spectrum, the shifts of the corresponding protons close to the ester group (Figures S1 and S2). Moreover, HRMS confirmed the formation of amphiphilic ligand **2** (see Figure S3).

The capacity of the oleyl 4-(dimethylamino) butanoate to self-assemble in micelles was determined by pyrene fluorescence assay. This method quantified the micelle formation threshold for the amphiphilic ligand, resulting in the critical micelle concentration (CMC). A CMC value of the amphiphilic ligand **2** was 0.24 mM (Figure S4), similar to the CMC values of low-molecular-weight amphiphiles described in the literature.^40,41^

**Figure 1:**
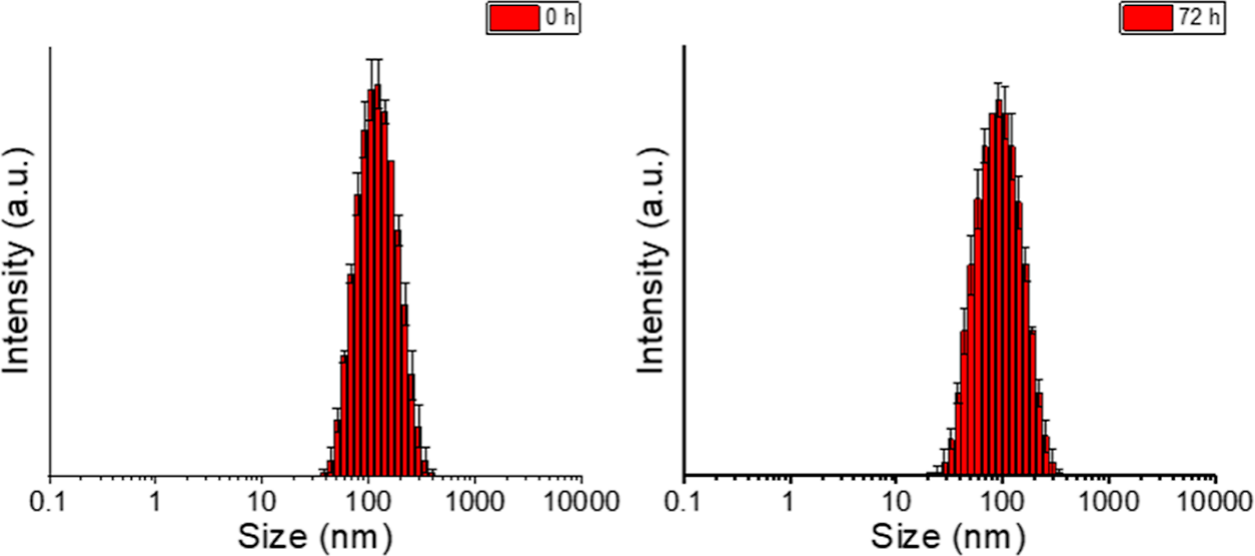
Size distributions by intensity of the tertiary amine-derived micelles at pH 6 (left: 0 h; right: 72 h).

First, the tertiary amine-derived micelles were characterized in water by DLS resulting micelles with an intensity size of 135.9 ± 3.6 nm and 0.20 of poly dispersity index (PDI). These results suggest that our amphiphilic ligand self-organized into monodisperse particles in water at pH 6 without the formation of aggregates. This behavior could be also observed over time, resulting in micelles with an intensity size of 101.8 ± 1.8 nm and 0.17 of PDI after 72 h (Figure 1). Furthermore, the stability of the formed micelles was evaluated under physiological conditions (PBS). The tertiary amine-derived micelles exhibited colloidal stability in physiological conditions for several days, with an intensity size of 109.7 ± 5.4 nm and 0.40 of PDI. Unfortunately, the visualization of the micelles by TEM did not produce clear images; it could be due to the low contrast from the organic supramolecular organization of the ligand.

### 3.2 pH Effect on the Tertiary Amine-Derived Micelles

**Figure 2:**
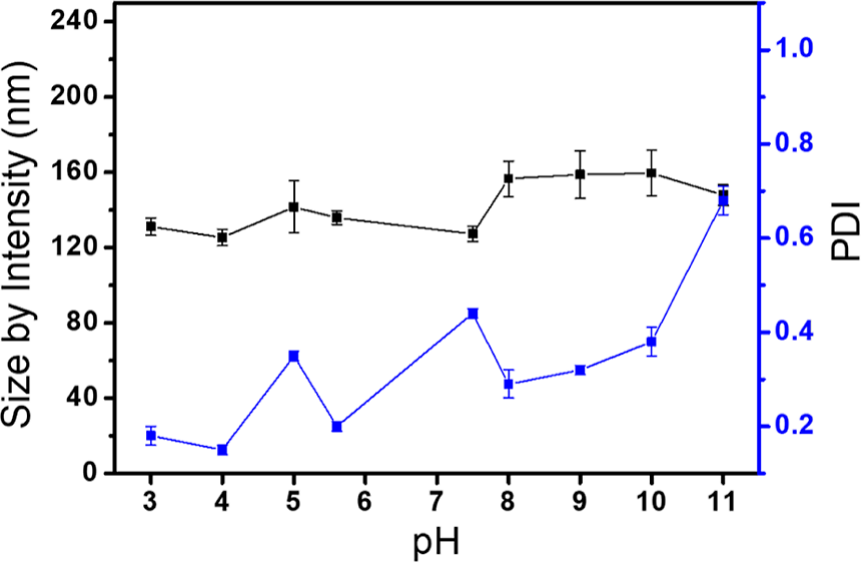
pH effect on tertiary amine-derived micelles in water.

The presence of tertiary amine moieties on the micelles is going to drive the colloidal stability of the self-organized ligands in function on the pH values, as commented in the Introduction Section. Therefore, the pH dependence of the micelle intensity size was performed by DLS. It was observed that when the pH of the micelle solution turned more acidic, the intensity size distribution remained relatively constant, with intensity size values of 137.9 ± 7.4 (PDI: 0.18) and 125.4 ± 4.4 nm (PDI: 0.15) at pH 3 and 4, respectively. It clearly indicates that the morphology and colloidal stability of tertiary amine-derived micelles remained unaltered from pH 6.8 to 3 due to the protonated amines and positive surface charge of the particles. On the other hand, at higher pH values, it means the pH of the micelles solution was more basic, and the tertiary amine moieties deprotonated with the consequence loss of surface charge, thus resulting in poly dispersed micelles with larger size distributions. As shown in Figure 2, the micelles at pH 7.5 exhibited a similar intensity size of 127.3 ± 4.0 nm to those at lower pH. However, the PDI value of these micelles was relatively higher, around 0.44, which indicates the appearance of a larger population of aggregates. Thus, at pH 8.1, it was observed an increase on the intensity size of the micelles to 156.6 ± 9.5 nm which was increased up to 170.1 ± 12.3 nm at pH 10 with a PDI value of 0.68. Therefore, it can be concluded that the change in the pH of the tertiary amine-derived micelles leads to the deprotonation of the corresponding amines, resulting in an increase in the intensity of the particles and a loss of colloidal stability. This effect is in accordance with the isoelectric points of similar tertiary amine moieties, which are in the pH range of 7.5–10.6. Hence, the pH effect on these micelles can be used for loading and releasing their payloads.

### 3.3 Incorporation of IONPs and AuNPs in the pH-Responsive Micelles

**Figure 3:**
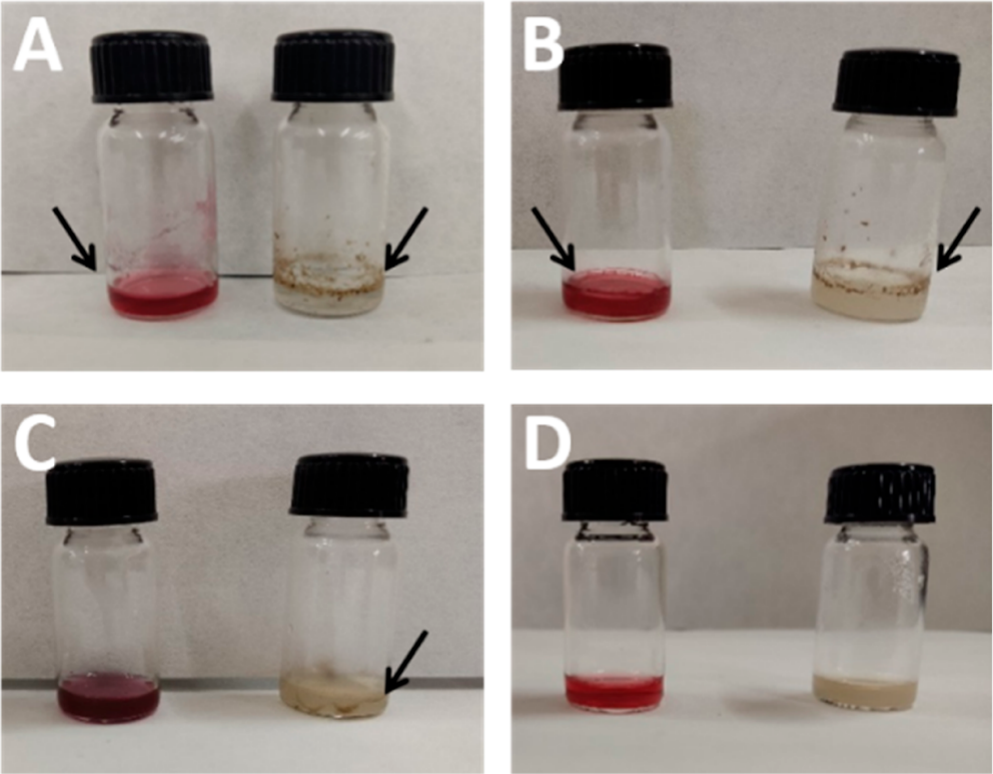
Photographs of the incorporation of inorganic NPs into the micelles (left: with AuNPs; right: with IONPs) by (A) evaporation at room temperature and pressure, (B) shaking, (C) sonication bath, and (D) probe sonicator. Arrows indicate precipitates.

The loading of nonwater-soluble inorganic NPs was conducted using various energy-based methodologies in order to obtain the best conditions for the formation of highly stable colloidal hybrid micelles. Both inorganic NPs, AuNPs and IONPs, were coated for a layer of hydrophobic surfactants, oleylamine and oleic acid, respectively. These hydrophobic coatings serve as a template for the self-assembly of the tertiary amine-derived ligand around the NPs, stabilizing the hydrophobic inorganic NPs in the medium. The critical step in the inorganic NPs loading into the micelles is the evaporation of the apolar solvent from the NP solution, hexane for both AuNPs and IONPs, forcing them to be encapsulated into the micelles. Due to the low boiling point of hexane, the initial attempt to load the inorganic NPs into the micelles was to simply leave a mixture containing an aqueous solution of the amphiphilic molecule **2** and a solution of the corresponding inorganic NPs in an uncapped vial. As shown in Figures 3A and S5, this procedure mainly transferred the AuNPs into the aqueous phase. However, this methodology also produced precipitates with both added inorganic NPs. It could be due to that the apolar solvent was not completely removed from the mixture. Therefore, a more energy-based procedure was applied. Then, shaking the mixtures in uncapped vials for 30 min yielded results similar to those obtained when the mixtures were simply left standing (Figures 3B and S6). Sonication in a sonication bath of the mixtures in capped vials for 30 min successfully transferred the hydrophobic AuNPs into the aqueous phase without the presence of precipitation. However, precipitates were still produced in the case of the IONPs (Figures 3C and S7). Finally, the application of ultrasonication for 30 min at mid power (55 W) to the mixtures using a probe sonicator rendered homogeneous colloidal solutions with both inorganic NPs, as can be observed in Figure 3D. By naked eye, it was clearly observed the absence of any precipitation or turbidity in the vials. The ultrasonic vibrations generated by the probe sonicator induced good dispersion of the NPs and also facilitated the rapid evaporation of hexane, which allowed for the self-organization of the micelles around the oleophilic-capped inorganic NPs. Hence, these results suggest that the slow evaporation of hexane and poor dispersion of the inorganic NPs in the mixture throughout the process were the likely causes of the issues with other procedures. Longer experimental times, by evaporation at room temperature and pressure or shaking methods, did not produce homogeneous colloid solutions, thus indicating the importance of the dispersion of the NPs in the solution during the experiment.

### 3.4 Characterization of the Inorganic NPs Loaded into the Tertiary Derived Micelles

DLS measurements, as shown in Table 1, demonstrated that the incorporation of the NPs into the micelles, by the probe sonicator method, produced monodisperse nanomaterials with PDI values similar to those of the empty tertiary amine-derived micelles (PDI: 0.20 at pH 6). So, in the case of the AuNP-loaded micelles, the hydrodynamic (HD) size was 113.9 ± 24.1 nm with a PDI of 0.29, and for the IONPs loaded-micelles, the HD size was 234.1 ± 13.4 nm with a PDI of 0.28 at pH 6 for both nanomaterials. In comparison with the HD size of the empty tertiary amine-derived micelles, the IONP-loaded micelles exhibited a significant increase in size, approximately 100 nm (a 73% enlargement). This increase in the diameter can be attributed to the size of the oleic acid-capped IONPs employed in the loading process.

**Table 1:** DLS Measurements of the Tertiary Amine-Derived Micelles

	intensity size distribution (nm) in water	PDI	intensity size distribution (nm) in PBS	PDI
empty micelles	135.9 ± 3.6	0.20	109.7 ± 5.4	0.40
AuNP-loaded micelles	113.9 ± 24.1	0.29	155.9 ± 3.7	0.49
lONP-loaded micelles	234.1 ± 13.4	0.28	182.4 ± 1.9	0.17

The hydrophobic IONPs probably served as a template for the self-organization of the tertiary amine-derived ligands, leading to an enlargement in the size of the resulting micelles. For the AuNPs, the template effect with respect to the size was not so important; the HD size distribution was quite similar to that of the empty micelles. However, since both inorganic NPs are capped with the same outer hydrophobic surface based on oleophilic surfactants such as oleyl amine and oleyl acid, for the AuNPs and IONPs, respectively, the diameter size of the inorganic NPs is the key factor for the final size of the loaded micelles. Indeed, the AuNPs exhibited a mean size of 9 nm, considerably smaller than the diameter size of the IONPs, 18 nm, as can been observed in the TEM images in Figures S8 and S9. Under physiological conditions, PBS, the HD size of the IONP micelles decreased to 182.4 ± 1.9 nm, representing a reduction of approximately 20%, similar to the reduction observed in the empty micelles. In contrast, the HD size of the AuNP-loaded micelles increased to 155.9 ± 3.7 nm. Although a decrease in the size was expected in a similar order to the other micelles, this result is in accordance with the enlargement size for the template effect previously obtained with the IONP-loaded micelles. In PBS, the HD size distribution of the AuNP-loaded micelles is notably narrower compared to those already reported in water (Table 1 and Figure S10). Therefore, this could be the reason for the tendency observed for AuNPs under physiological conditions.

**Figure 4:**
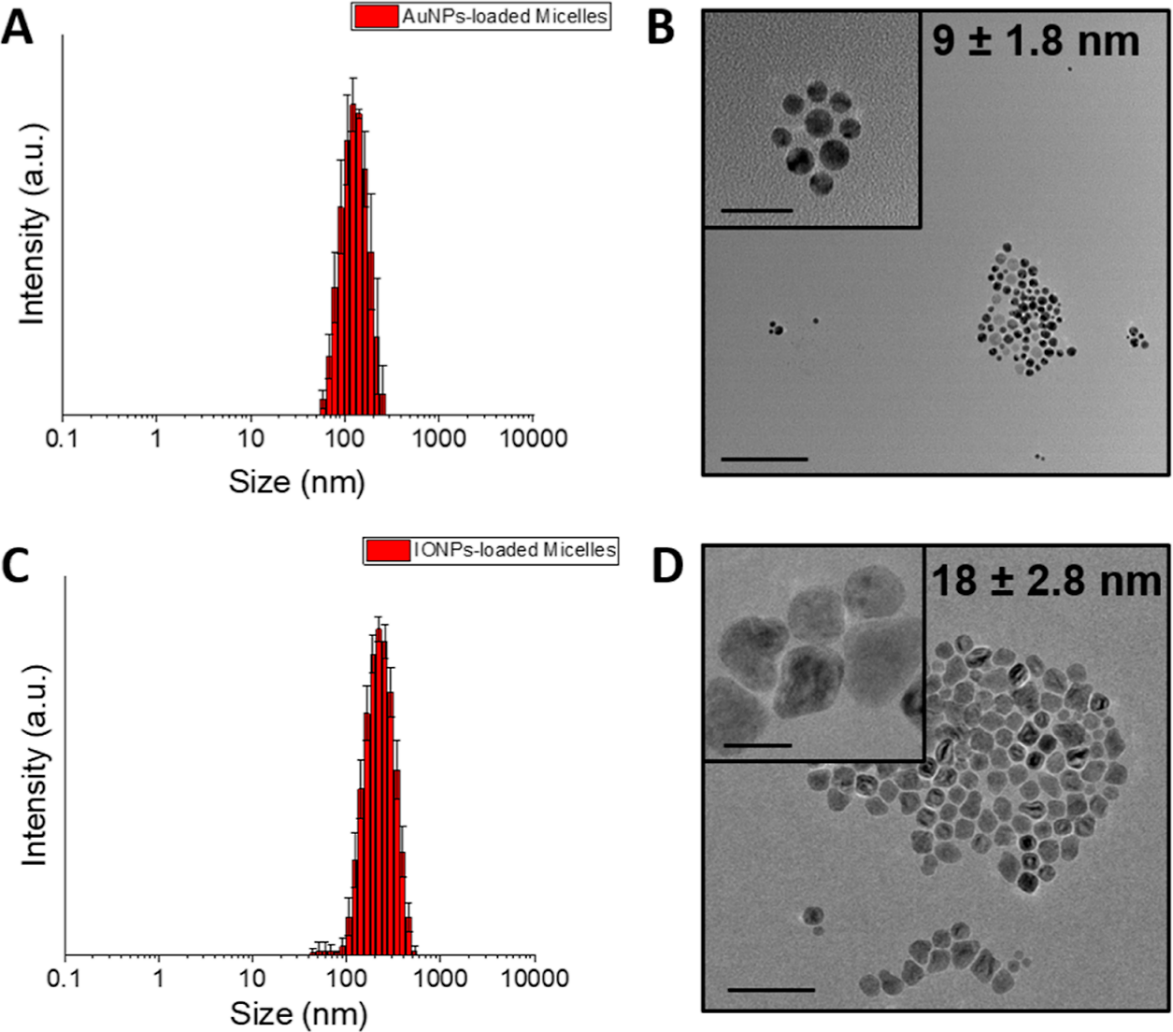
(A) Size distribution by intensity of the AuNP-loaded micelles at pH 6; (B) representative TEM images of the AuNP-loaded micelles in water. Scale bars correspond to 100 nm for the low-magnification TEM image and 25 nm for the inset. (C) Size distribution by intensity of the IONP-loaded micelles at pH 6; and (D) representative TEM images of the IONP-loaded micelles in water. Scale bars correspond to 100 nm for the low-magnification TEM image and 25 nm for the inset.

TEM images confirmed the presence of inorganic NPs in aqueous solution. However, the TEM just showed the AuNPs and IONPs due to the higher contrast compared to the tertiary amine-derived micelles (Figure 4). Nevertheless, the TEM images depicted homogeneous and well-dispersed inorganic NPs loaded into the micelles.

**Figure 5:**
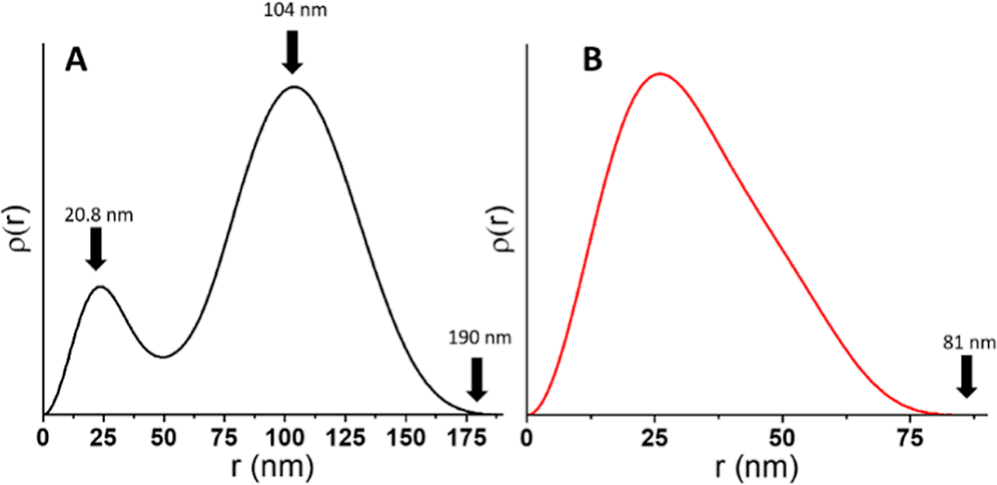
Normalized pair distribution of the SAXS measurements versus distance graphs of (A) IONP-loaded micelles and (B) AuNP-loaded micelles.

Small-angle X-ray scattering (SAXS) studies of the inorganic NP-loaded micelles were evaluated to confirm the formation of these nanostructures. The analysis demonstrated that the maximum distance (*r*) values in the graphs correspond to the size of the NP-loaded micelles, 81 and 190 nm for the AuNP- and IONP-loaded micelles, respectively (Figures 5 and S11). However, the HD sizes of these nanostructures in water determined by DLS exhibited higher values due to the influence of the water that is suppressed in the SAXS analysis. As shown in Figure 5A, the main population at 104 nm indicates the thickness of the organic shell of the IONP-loaded micelles. This distribution displays the radius of the organic shell and the inorganic IONP core (Figure S12). Therefore, the size of the organic layer was determined to be 83 nm. Moreover, the other population at 18 nm could correspond to the scattering of the oleic acid-capped IONPs. However, in the case of AuNP-loaded micelles, it was not possible to determine the thickness of the organic shell due to the overlapping of the populations (Figure 5B).

**Figure 6:**
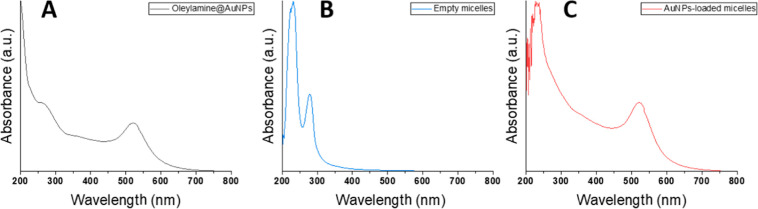
UV–vis absorption spectra of (A) oleylamine-capped AuNPs in hexane; (B) empty micelles in water; and (C) AuNP-loaded micelles using the probe sonicator method in water.

Optical properties of the inorganic NP-loaded micelles were analyzed by UV–vis absorption spectroscopy. The AuNP-loaded micelles exhibited a typical surface plasmon resonance band centered at 522 nm in water at pH 6, which is almost identical to that of 9 nm oleylamine-capped AuNPs in hexane (Figures 6 and S13). These results indicate the absence of aggregated NPs, which agree with the TEM observations. For the IONP-loaded micelles, UV–vis absorption spectroscopy did not show significant results (Figure S14).

**Figure 7:**
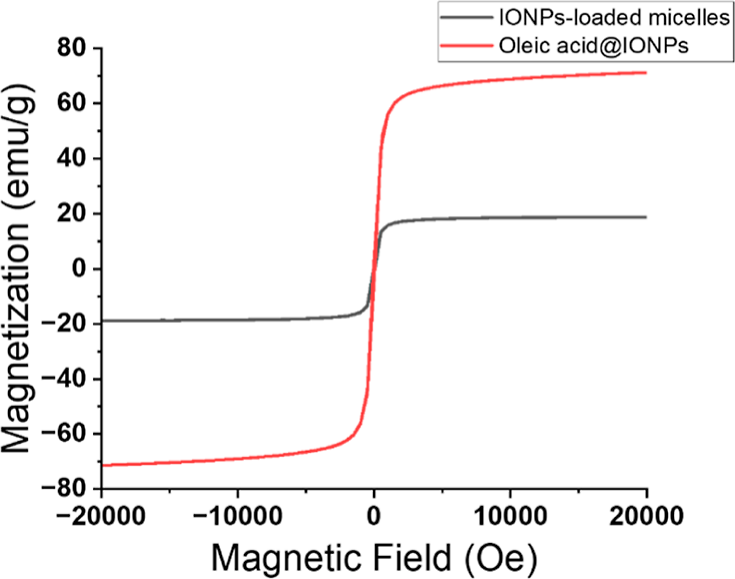
Hysteresis loops of oleic acid-capped IONPs (red) and IONP-loaded micelles (black).

The magnetic properties of the aqueous colloidal IONP-loaded micelles were analyzed by using a vibrating sample magnetometer at room temperature. The magnetic measurement of the IONP-loaded micelles showed similar superparamagnetic features, coercivity and remanence magnetization ≈0, to the hydrophobic oleic acid-capped IONPs (Figure 7). In the case of the saturation magnetization (Ms), the IONP-loaded micelles exhibited a value of 18 emu/g, slightly lower than the initial hydrophobic IONPs (Ms = 71 emu/g). This decrease in the Ms value is attributed to the thicker organic coating that encapsulated the IONPs.

### 3.5 pH Effect on the Hybrid Micelles

**Figure 8:**
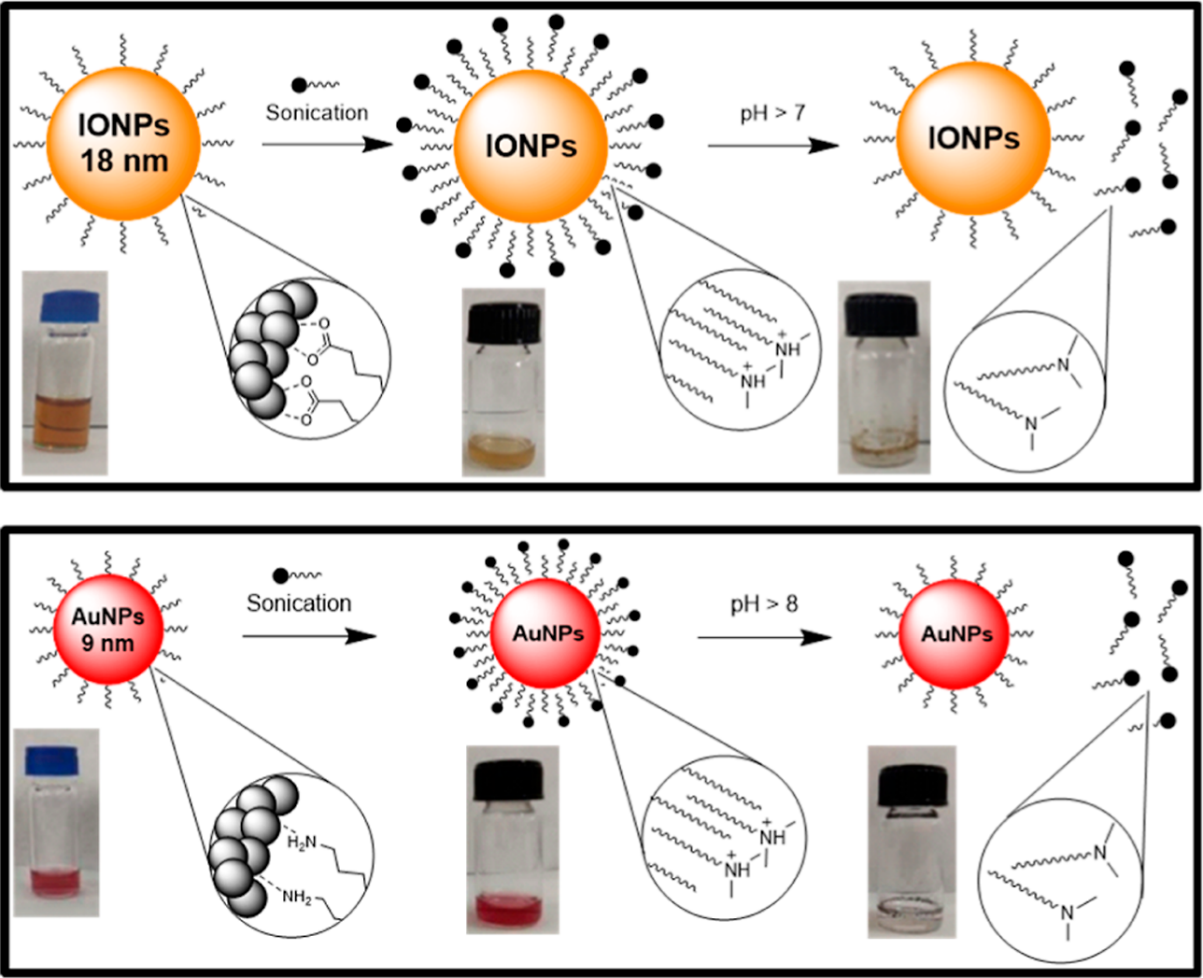
Schemes of the incorporation and release processes of 18 nm IONPs (top) and 9 nm AuNPs (bottom) from the pH-responsive micelles.

**Figure 9:**
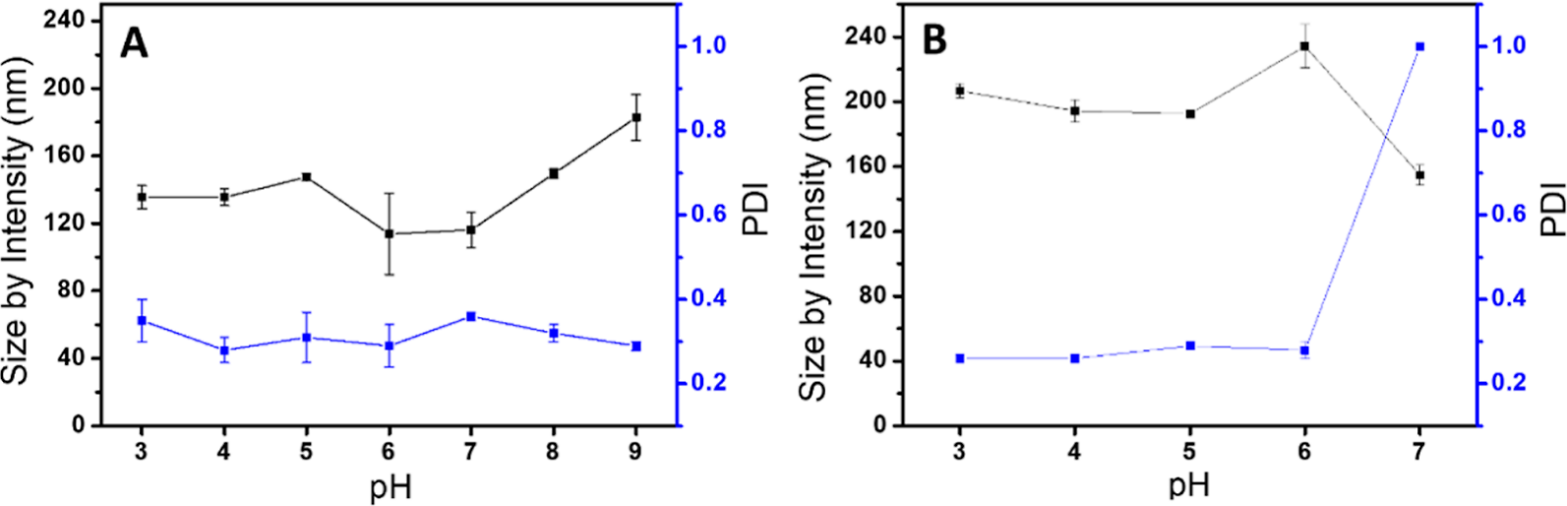
Effect of pH on hydrodynamic sizes in water of AuNPs- (A) and IONP-loaded micelles (B).

To investigate the pH effect of these hybrid tertiary amine-derived micelles, we studied both as-prepared micelles loaded with the inorganic NPs by DLS (Figure 8). The colloidal solutions of AuNP- and IONP-loaded micelles remained relatively stable, with slight variations in the HD sizes when the pH of the medium was in the range between 3 and 7 (Figure 9). For instance, the AuNP-loaded micelles exhibited no precipitation within this pH range, with a minor enlargement in the HD size from 116.2 ± 10.5 nm at pH 7 to 135.6 nm ± 6.9 nm at pH 3. It is worth noting that the size remained constant at pH 4 and 3. Remarkably, the PDI values of the AuNP-loaded micelles followed a similar trend to the HD sizes when the pH was adjusted to acidic pH, with PDI values around 0.30. At pH 3, a higher PDI value of 0.35 was observed in the AuNP-loaded micelles, indicating the beginning of the appearance of polydispersity. This change can probably be attributed to the hydrolysis of the ester groups within the tertiary amine-derived micelles, which could trigger the collapse of the micelles over time. At pH higher than 7, the HD size of the AuNP-loaded micelles started to increase to 182.8 nm before the appearance of the precipitation of the oleyl amine-capped AuNPs. On the other hand, the IONP-loaded micelles exhibited a similar behavior against the pH. Within the pH range 3–6, the HD sizes of the IONP-loaded micelles were in the same order, with minor variations, ranging between 234.3 nm ± 13.4 nm at pH 6 to 206.7 ± 4.4 nm at pH 3, and exhibited PDI values below 0.29. On the contrary, at pH 7, the HD sizes of the IONP-loaded micelles exhibited a usual reduction of their size to 154.8 nm ± 6.2 nm with a PDI value of 1. This PDI value indicates clearly that the IONP-loaded micelles began to precipitate at pH lower than the AuNP-loaded micelles. In fact, it was not possible to measure the HD sizes of the IONP-loaded micelles at pH higher than 7 due to the complete precipitation of the NPs. Therefore, the higher stability of the AuNP-loaded micelles is likely attributed to the smaller size of the hydrophobic AuNPs in comparison to the IONPs. Indeed, the size of the inorganic NPs plays a significant role in the hydrophobic/hydrophilic balance of the hybrid micelles. The larger IONPs required more hydrophilic ligands to render stable micelles. Consequently, an increase in pH causes a deprotonation of the tertiary amine moieties of the micelles, which reduces their hydrophilicity. This can lead to the precipitation of the hybrid micelles and the concomitant release of the hydrophobic IONPs.

### 3.6 In vitro Release Studies of (S)-Camptothecin

**Figure 10:**
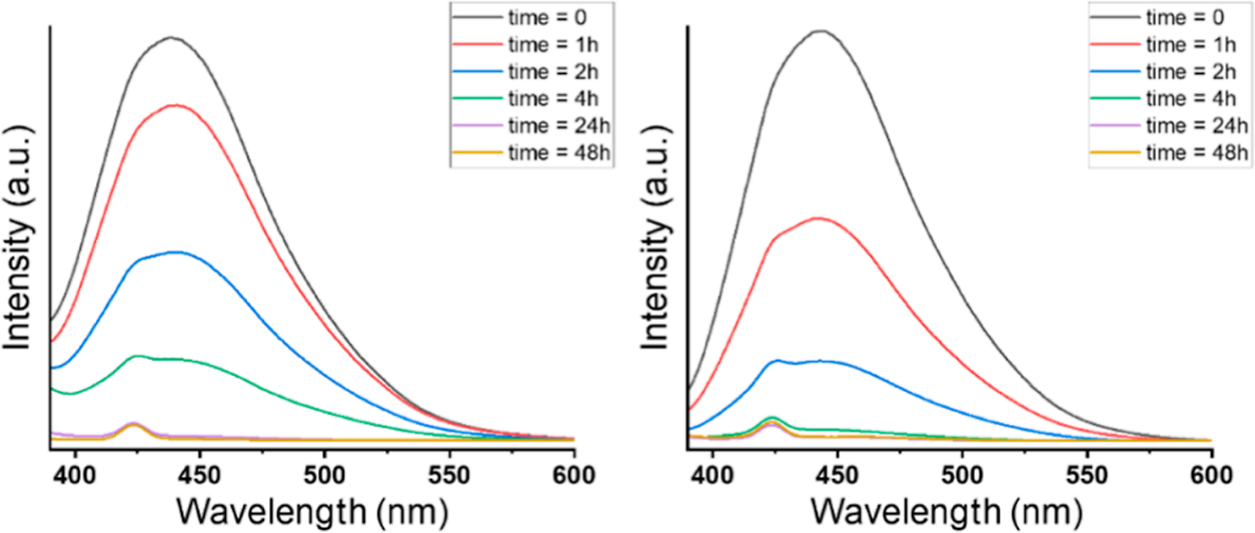
In vitro release study of camptothecin-loaded micelles. Plot of fluorescence in MES buffer (0.5 M) at pH 6 (left) and PBS (1×) at pH 7.2 (right) vs time.

To confirm the potential of our pH-responsive micelles, in vitro release studies have been carried out using CPT as a hydrophobic model drug. CPT is a well-known potent antiproliferative compound against different cancer cells.^42^ However, one of the main issues with CPT for clinical practice is its lack of solubility in water. Therefore, the sonication of CPT in the presence of the amphiphilic molecule **2** in water resulted in the formation of stable CPT-loaded micelles. After centrifugation, the nonloaded CPT was removed, and the presence of CPT in the micelles was evaluated by DLS and fluorescence. DLS characterization resulted in CPT-loaded micelles with smaller sizes than the empty micelles, with an intensity size of 115.0 ± 0.4 nm and a PDI value of 0.18 in water at pH 6. In 2-(*N*-morpholino)ethanesulfonic acid (MES) buffer, 0.5 M at pH 6, the HD size decreased slightly to 109.6 ± 4.7 nm with a PDI value of 0.2. However, in PBS, 1× at pH 7.2, the CPT-loaded micelles exhibited HD sizes >1000 nm with a PDI value of 0.8, indicating the presence of aggregates. The fluorescence intensity of the CPT-loaded micelles using wavelength excitation of 370 nm exhibited an emission band around 450 nm. This clearly evidences the success of the drug encapsulation into our micelles. Subsequently, the release of CPT at different pH values was carried out to confirm the responsiveness of our nanosystems. MES buffer at 0.5 M and pH 6, and PBS at 1× at pH 7.2 were the saline solutions utilized in this study. Both buffers exhibited different release behaviors, as can be observed in Figure 10. The fluorescence decay was more prominent in PBS than that in the MES buffer. After 4 h, CPT was practically released from the micelles in PBS, but it was still present in the micelles in the MES buffer. Finally, after 24 h, CPT was also released from the micelles at pH 6.

## 4 Conclusions

In summary, we successfully synthesized pH-response hybrid micelles by combining inorganic NPs with tertiary amine-derived micelles. The synthesis of the micelles precursor was performed in a one-pot synthesis from oleyl alcohol and an acyl chloride, which presents a tertiary amine group as a pH response moiety. The tertiary amine-derived precursor readily formed micelles at neutral-acidic pH conditions with a CMC of 0.24 mM. At a basic pH of 10 or higher, the empty micelles began to disassemble, likely due to the loss of hydrophilia of the amphiphiles induced by the deprotonation of the tertiary amine moieties. The preparation of hybrid colloidal micelles was developed with two different sizes of gold NPs and iron oxide NPs. These NPs are of interest in the nanomedicine field as contrast agents and hyperthermia agents for the more efficient diagnosis and treatment of diseases. However, we propose these inorganic NPs as model payloads that could be extended to other hydrophobic inorganic NPs. The hydrophobic AuNPs and IONPs used in this study were capped with oleophilic surfactants, which acted as templates to facilitate the self-organization of the tertiary amine-derived ligands in micelles through hydrophobic interactions between the oleophilic coating and the oleyl derived ligands. This process led to water-soluble colloidal solutions of the inorganic NPs incorporated within the micellar structures, triggered by the application of ultrasonication with a probe sonicator. The pH responsiveness of the synthesized hybrid micelles could be tuned by adjusting the size of the inorganic NP payloads. In the case of smaller inorganic NPs with diameter sizes of 9 nm, the micelles exhibited a pH response above pH 8. For larger inorganic NPs with sizes of 18 nm, the pH response occurred at a lower pH, above pH 7. This effect can be attributed to the different hydrophilic/hydrophobic balances of the hybrid micelles. Remarkably, larger inorganic NPs required a greater proportion of hydrophilia to render water stable nanomaterials. Moreover, a hydrophobic drug, CPT, has been encapsulated within the tertiary amine-derived micellar structures to assess their potential as nanosystems for drug release. In vitro release studies carried out in the resulting CPT-loaded micelles, at two different pH, exhibited a faster release of CPT at pH 7.2 compared to pH 6. This is in agreement with results obtained from the inorganic NP payloads and further validates the efficacy of our pH-response micelles in releasing hydrophobic drugs. Therefore, these results open a new way for the synthesis of tunable pH-responsive hybrid micelles with great potential for both in vitro and in vivo biomedical imaging, hyperthermia treatment, and also drug delivery nanosystems.

## Supporting Information Available

NMR spectra, HRMS, CMC graph, size distribution graphs, TEM images, SAXS measurements, and UV–vis spectra of the IONPs and AuNPs (PDF)
