# Supplementary material for: Efficient Strategy to Synthesize Tunable pH-Responsive Hybrid Micelles Based on Iron Oxide and Gold Nanoparticles
Source: Langmuir. 2024 May 20;40(22):11775–84. doi: 10.1021/acs.langmuir.4c01318 (PMC11155236; doi:10.1021/acs.langmuir.4c01318)
Supplement: Supplementary file 1 — la4c01318_si_001.pdf [file la4c01318_si_001.pdf]

Supporting Information

Efficient Strategy to Synthesize Tunable pH-Responsive Hybrid Micelles Based on Iron Oxide and Gold Nanoparticles.

Raúl Gimeno-Ferrero,[a] Javier Rodríguez de Jesús[a] and Manuel Pernia Leal.*[a]

[a]*Departamento de Química Orgánica y Farmacéutica, Facultad de Farmacia, Universidad de Sevilla, c/ Profesor García González, 2, 41012 Sevilla, Spain.*

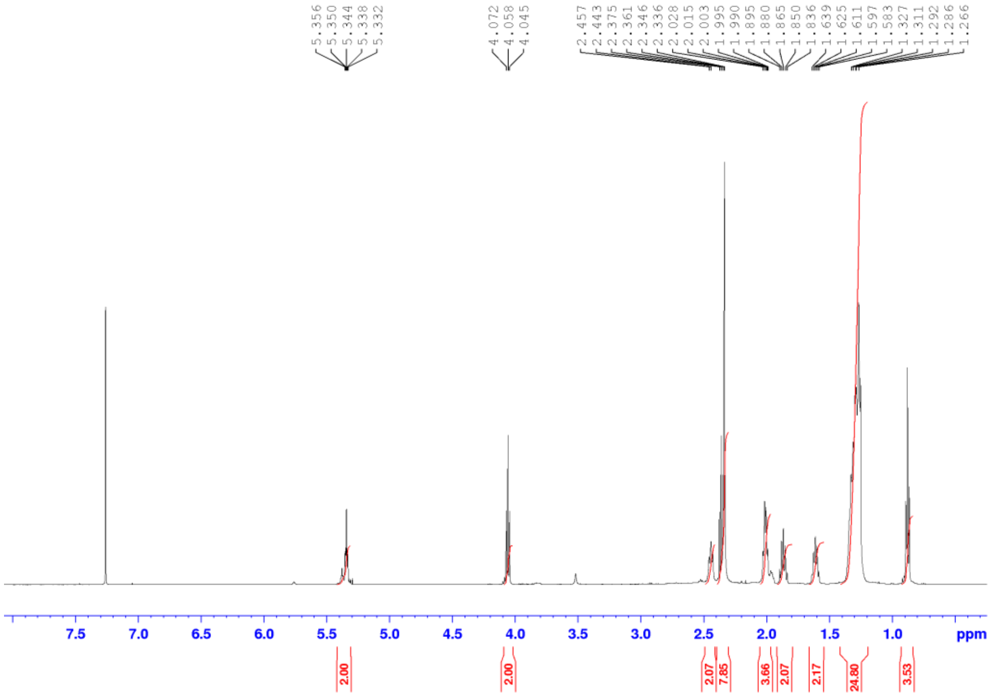

Figure S1. ^{1}H NMR spectrum of **2** in $CDCl_3$.

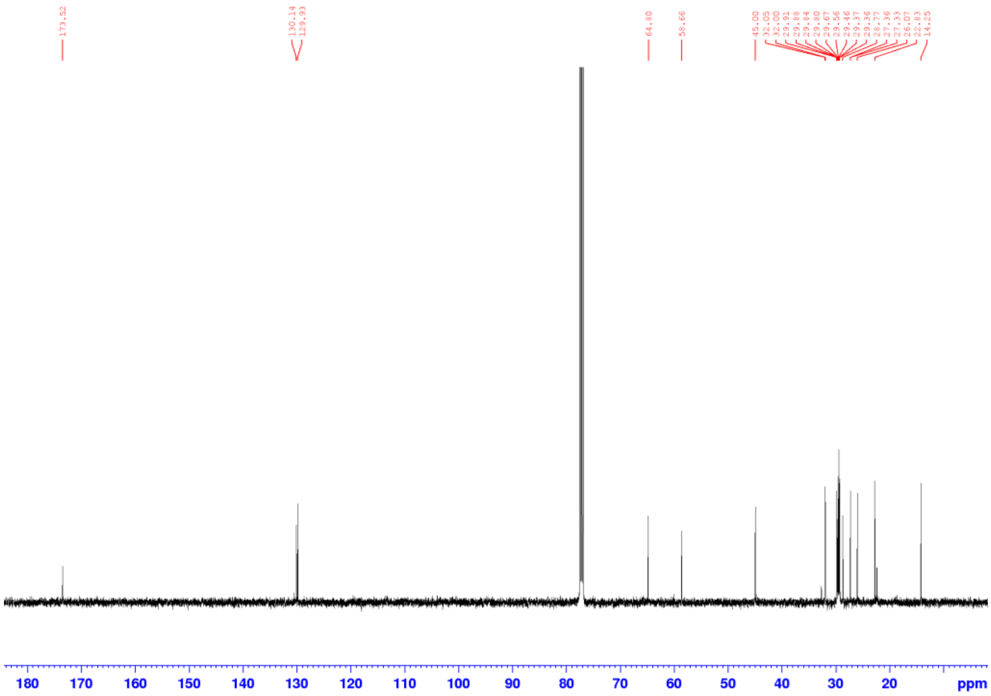

Figure S2. ^{13}C NMR spectrum of **2** in $CDCl_3$.

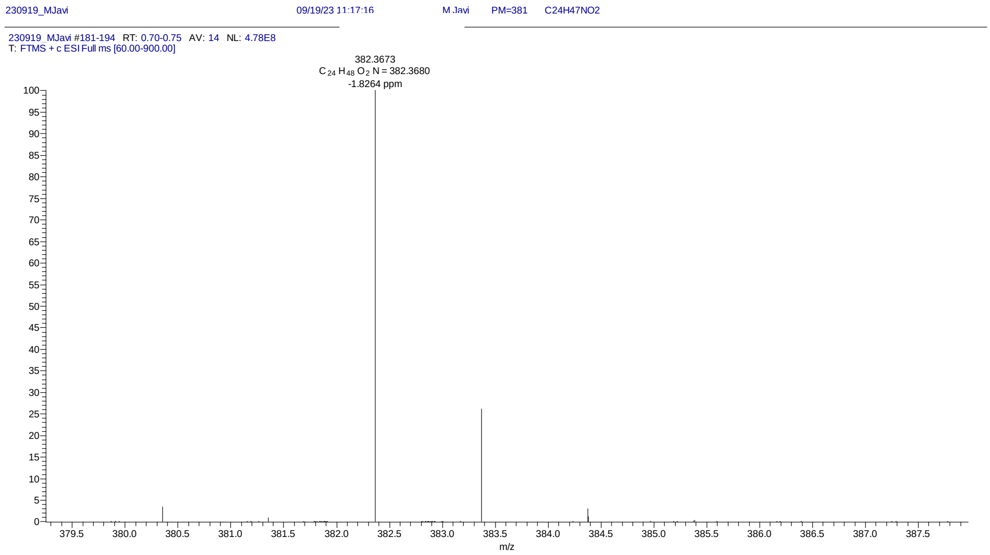

Figure S3. High resolution mass spectrum of **2**.

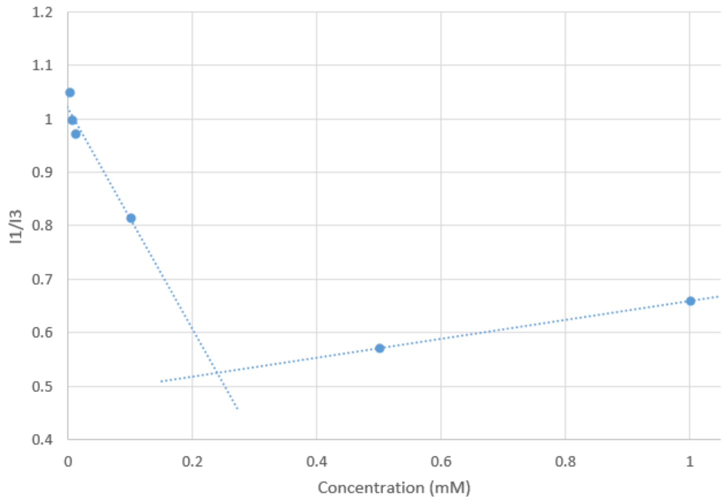

Figure S4. Representation of I1/I3 vs amphiphile **2** concentration.

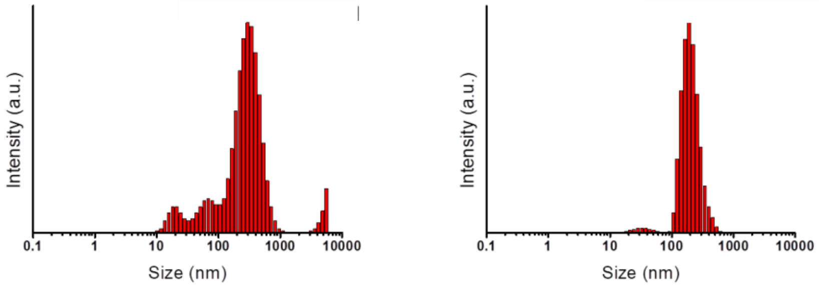

Evaporating at rt and pressure Method	PDI	Size (nm)
AuNP-loaded micelles	0.52	295.3
IONP-loaded micelles	0.21	255.0

Figure S5. Size distributions by intensity of AuNP-loaded micelles (Left) and IONP-loaded micelles (Right) produced by the evaporation method, and their DLS data (Down).

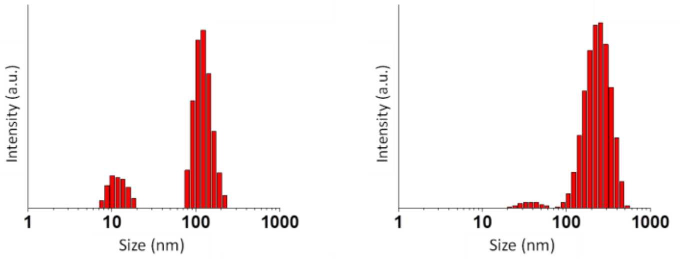

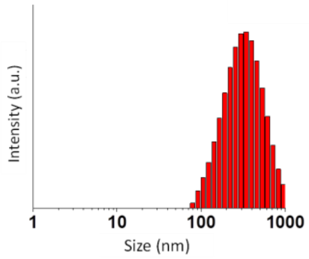

Shaking Method	PDI	Size (nm)
Empty micelles	0.28	122.4
AuNP-loaded micelles	0.39	255.0
IONP-loaded micelles	0.24	342.0

Figure S6. Size distributions by intensity of empty micelles (top right), AuNP-loaded micelles (top left) and IONP-loaded micelles (bottom left) produced by the shaking method; and their DLS data (bottom right).

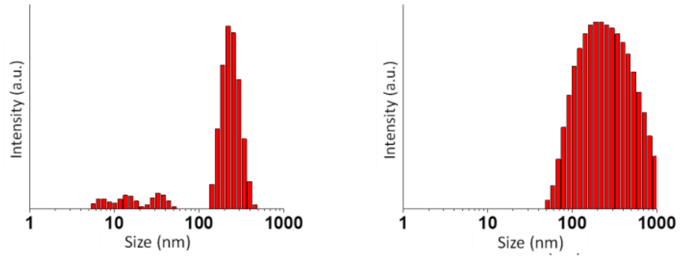

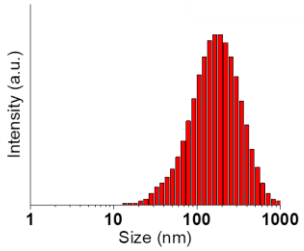

Sonication Bath Method	PDI	Size (nm)
Empty micelles	0.33	220.2
AuNP-loaded micelles	0.42	190.1
IONP-loaded micelles	0.31	164.2

Figure S7. Size distributions by intensity of empty micelles (top right) AuNP-loaded micelles (top left) and IONP-loaded micelles (bottom left) produced by the sonication bath method; and their DLS data (bottom right).

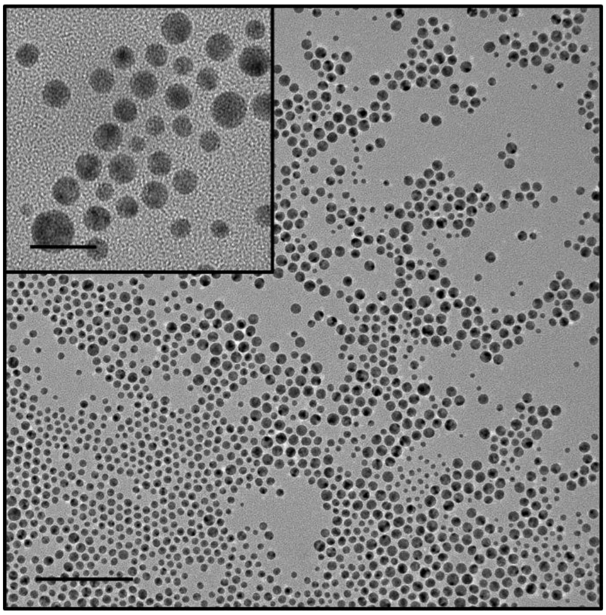

Figure S8. Representative TEM images of 9 nm oleylamine-capped AuNP in hexane. Scale bars correspond to 200 nm for the low magnification TEM image and 50 nm for the inset.

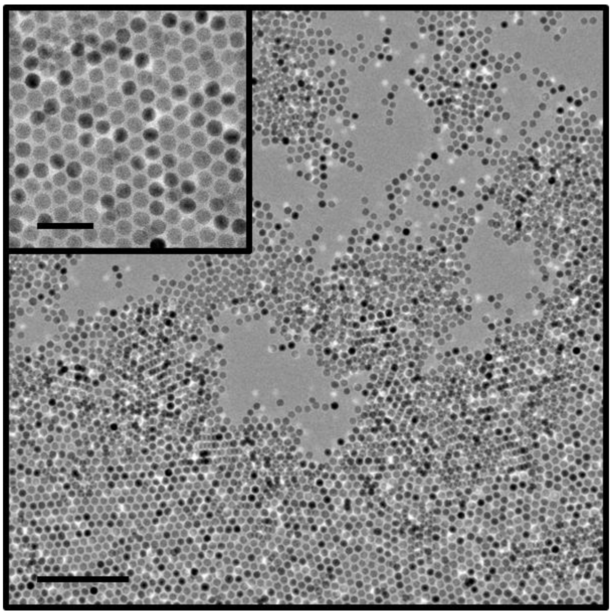

Figure S9. Representative TEM images of 18 nm oleic acid-capped IONP in toluene. Scale bars correspond to 200 nm for the low magnification TEM image and 50 nm for the inset.

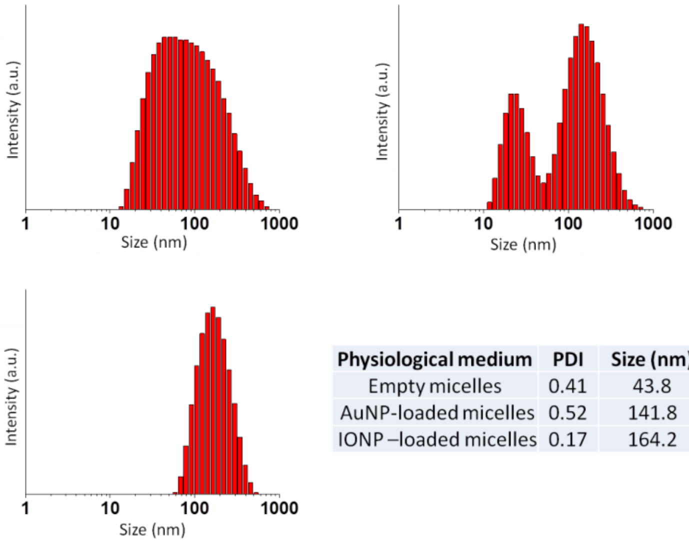

Physiological medium	PDI	Size (nm)
Empty micelles	0.41	43.8
AuNP-loaded micelles	0.52	141.8
IONP –loaded micelles	0.17	164.2

Figure S10. Size distributions by intensity of empty micelles (top right), AuNP-loaded micelles (top left) and IONP-loaded micelles (bottom left) in physiological medium; and their DLS data (bottom right).

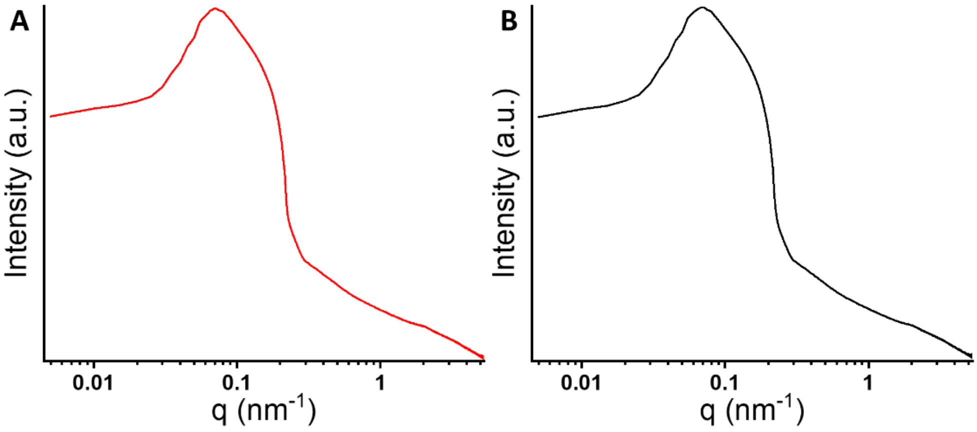

Figure S11. SAXS measurements of (A) AuNP-loaded in micelles, and (B) IONP-loaded in micelles.

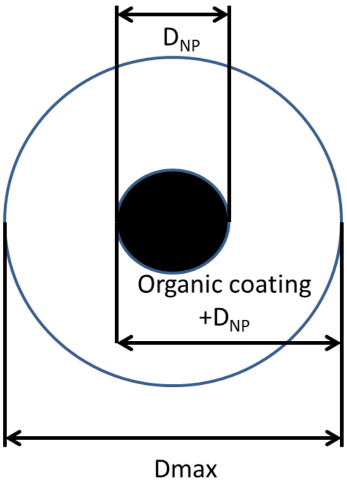

Figure S12. Distances measured in the nanostructures with the SAXS measurements.

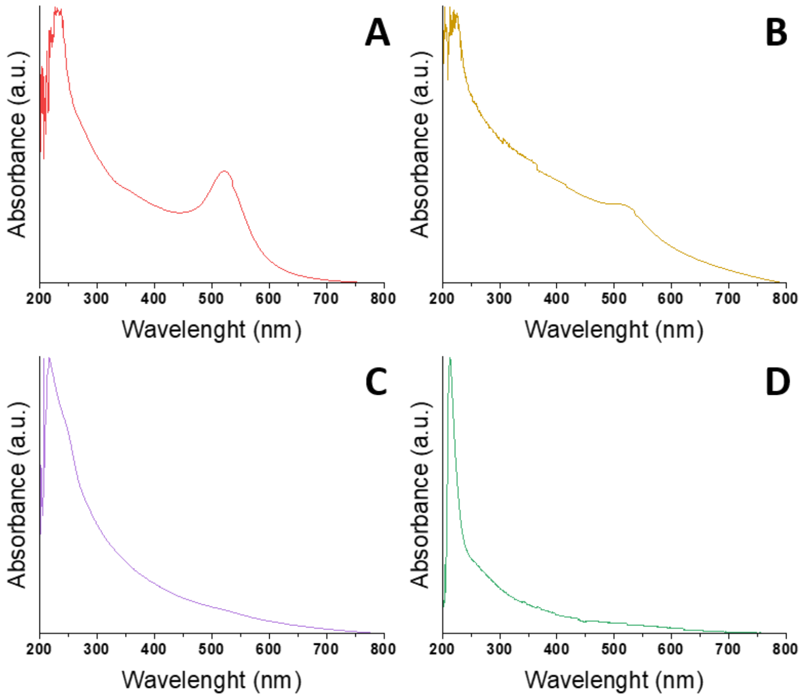

Figure S13. UV-Vis absorption spectra of AuNP-loaded micelles by different methods: (A) probe sonicator; (B) sonication bath; (C) shaking and (D) evaporation at room temperature and pressure.

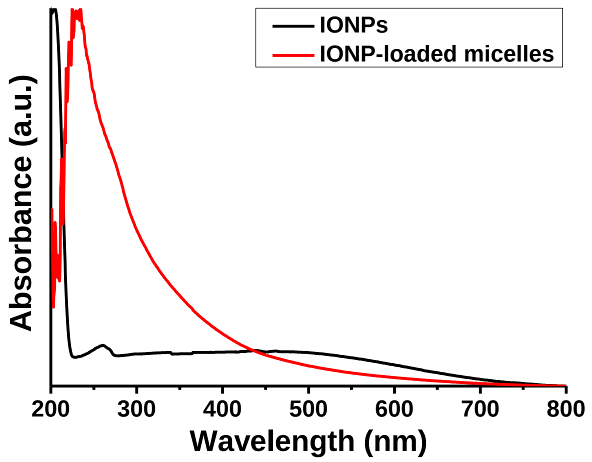

Figure S14. UV-Vis absorption spectra of oleic acid-capped IONP in toluene and IONP-loaded micelles in water at pH 6.
